# Injuries Due to Law Enforcement Use of Force in the United States, 2006-2015: Trends in Severity and by Race

**DOI:** 10.1007/s40615-023-01733-z

**Published:** 2023-08-08

**Authors:** E. M. F. Strömmer, Wendy Leith, Maurice P. Zeegers, Michael D. Freeman

**Affiliations:** CAPHRI School for Public Health and Primary Care, Faculty of Health, Medicine, and Life Sciences, Maastricht University, Maastricht, Netherlands

**Keywords:** Use of force, Race, Law enforcement, Emergency department, Epidemiology, Injury

## Abstract

**Purpose:**

The purpose of the study was to assess incidence and severity of hospital reported injuries related to law enforcement Use of Force (UoF) in the US over time, and by race.

**Methods:**

Data from the National Emergency Department Sample from the Healthcare Cost and Utilization Project and the National Electronic Injury Surveillance System (NEISS-AIP) from US Consumer Product Safety Commission were queried to identify UoF injuries. Regression analysis, t-tests, and chi-square tests were used in the analysis.

**Results:**

Between 2006-15, there were 529,259 emergency department admissions for UoF injury in the NEDS, and 870,779 admissions in the NEISS-AIP. In a model adjusting for year, sex, and age, the Injury Severity Score increased by 1.1% annually (*p<*0.0001). Black people were 6 times as likely to be admitted to the ED as White people or Native Americans/Alaska Natives, and 25 times as likely as Asians/Pacific Islanders. Black patients were 4 times as likely as White patients to be admitted as inpatients. Per arrest rate by race using FBI uniform crime reporting data, Black arrestees were 2.5-3.1 times as likely as any other race to be hospitalized for UoF injury.

**Conclusion:**

The results of the study demonstrate that US law enforcement are injuring civilians more frequently and severely over time, and that Black people are disproportionately affected.

## Introduction

Law enforcement officers are legally authorized to use physical force as a means of controlling the movement of suspects. Use of force (UoF) is a term reserved for law enforcement or other authority-related intervention, including correctional officers and security guards, and tactics range in severity from verbal commands to non-lethal and intentionally lethal methods. Public interaction with law enforcement is common in the US, with around 1 in 10 people in the population (more than 31 million) having contact with law enforcement in a given year [1]. An estimated 1-2% of law enforcement interactions involve UoF [2, 3], with Black and Hispanic people experiencing higher rates of both non-lethal and lethal UoF than White people, per interaction [2–7].

There was no publicly available national surveillance system for tracking law enforcement UoF-related fatalities or injuries in the US until after the death of George Floyd in 2020, when the Federal Bureau of Investigations (FBI) published participation data from a database launched in 2019 of law enforcement UoF events resulting in death or serious injury, or involving firearm discharge in the direction of a person [8]. The FBI National Use-of-Force Data Collection database does not provide a comprehensive representation of UoF in the US, however: because agency participation in the database is voluntary, the data are representative of less than half of law enforcement agencies in the nation as of 2020, and are inherently subject to self-reporting bias. As of 2023, only participation data have been published; as more agencies participate, the FBI will report responses, percentages, and ratios relating to UoF events. National estimates of UoF-associated mortality are reported by the Centers for Disease Control and Prevention (CDC) and detailed in the Multiple Cause of Death (MCOD) database. According to MCOD data, there were more than 10,000 deaths attributed to law enforcement interaction between 1999 and 2020, or approximately 500 deaths per year [9]. In contrast with this estimate, Fatal Encounters, a nonprofit organization that collects and analyzes information about law enforcement-related deaths from media accounts, estimates that the annual number of deaths secondary to UoF by law enforcement is approximately 2,000, as of 2020 [10]. The explanation for this difference is likely related to difficulties with surveillance: an analysis that compared the US government-published database of vital registration (the National Vital Statistics System [NVSS]) with non-governmental databases of law enforcement-related deaths reported that more than half of law enforcement-related deaths were not reported in the NVSS [11]. The analysis also concluded that mortality from law enforcement interaction was highest for Black males, versus other races and sexes, and that deaths from law enforcement interaction increased by 38% between 1980 and 2019. The evidence indicates that the failure of US government databases to accurately track the number of law enforcement-related deaths is a racially disparate public health issue that is getting worse with time.

Surveilling for law enforcement UoF-related injuries is even more difficult than tracking deaths, as survivable injuries resulting from encounters with law enforcement are not necessarily followed by administrative investigation or medical record keeping, much less media reports. The most reliable research has used hospital data to estimate the frequency of UoF-related injury. A study of law enforcement-related injuries over time by Kaufman et al. identified an annual average of 51,000 emergency department (ED) encounters for law enforcement-related injuries from 2006-12 using the Nationwide Emergency Department Sample (NEDS) database from the Healthcare Cost and Utilization Project (HCUP) of the Agency for Healthcare Research and Quality (AHRQ) [12]. Across the 7 years studied, the frequency of ED admission for law enforcement-related injury was stable, and the most common type of injury was from being struck (*e.g.,* by fist or baton). The analysis did not evaluate or account for race, however, as this variable was not coded in NEDS data until 2019.

In 2017, Miller et al. used multiple US national databases to characterize injury and death from law enforcement UoF for a single year, while accounting for racial disparities. The study included data from vital statistics, media reporting, FBI arrest data, police public contact surveys, and HCUP ED and inpatient databases [13]. The authors identified reports of 55,400 people injured or killed by law enforcement in 2012 and found that 1 in every 291 (0.3%) law enforcement arrests or stops resulted in hospital-treated injury or death. Black, Hispanic, and Native American people were stopped or arrested at higher rates per population than White or Asian people.

A study by Feldman et al. analyzed the frequency of law enforcement-related injuries in EDs across 15 years, accounting for race. The study authors identified 683,033 reports of law enforcement-related injury in men and women aged 15-34 from 2001-14 using the National Electronic Injury Surveillance System - All Injuries Program (NEISS-AIP), a database produced by the US Consumer Product Safety Commission and Centers for Disease Control and Prevention [14]. The authors reported an annual increase in the rate of admission for law enforcement-related injury and concluded that Black people were nearly 5 times as likely to experience injury from interaction with law enforcement than White people.

In the current study, we aim to expand upon the existing body of knowledge regarding law enforcement-related injury rates by examining not only the incidence, but also the severity of law enforcement-related injuries over time, as well as the effect of race on the incidence and severity of injury. Due to the limitations of publicly available databases of hospital-reported injuries in the US, the proposed study will analyze data from both the NEDS and the NEISS-AIP.

## Materials and Methods

The proposed study was analyzed in two parts, using two different publicly available national databases. Each analysis is described below:

### 2006-2015 NEDS: National Sample of Emergency Department Visits

The National Emergency Department Sample (NEDS) is made available through the Healthcare Cost and Utilization Project (HCUP) of the Agency for Healthcare Research and Quality of the US Department of Health and Human Services [15]. The NEDS is a nationally representative stratified sample of hospital emergency department (ED) records from the United States. Produced annually, it comprises approximately 30 million discharge records from 953 hospitals in 36 states, and represents 20% of all ED discharges. The sample is weighted to provide a national estimate of ED admission characteristics. The NEDS provides information on patient demographics, up to 15 diagnoses, up to 15 procedures, up to 4 external cause codes (“E-codes,” which designate mechanism and/or intent of injury), admission and discharge status, payer characteristics (*e.g.,* private versus public insurance) and total ED charges.

The 2006 – 2015 NEDS databases were queried for all records with an external cause code (E-code) indicating legal intervention, meaning that the injury was sustained as the result of an encounter of an interaction with any law enforcement official, on- or off-duty. Records with E-codes for late effects of legal intervention and legal execution were excluded. The E-codes were divided into the following categorical variables: firearms or explosives, gas or poisoning, and manhandling, cut, or blunt object. Categories were grouped according to potential lethality (*i.e.* pepper spray [gas] is on the less-lethal end of the UoF spectrum, while firearms and explosive are more lethal forms of UoF).

For years 2009 – 2014 (Q1 – Q4) and 2015 (Q1 – Q3), the NEDS databases include an Injury Severity Score (ISS). The ISS is a standardized cumulative score for traumatic injury, which consists of the sum of the squares of the highest Abbreviated Injury Scale (AIS) scores in 3 out of 6 body regions, for a possible total of scale 1-75 [16]. A higher score indicates more severe injury, and a score of 75 indicates non-survivable injury. For years 2009-2015, HCUP assigned each record an ISS using the International Classification of Diseases Program for Injury Categorization (ICDPIC) [17, 18]. The ICDPIC was used in the present study to assign an ISS for each record for years 2006-2008, as ISS was not calculated by HCUP for these years. The fourth quarter of 2015 was excluded in the analysis as it utilizes the ICD-10 (rather than the ICD-9), and inclusion would have increased the potential for information bias. Records with missing or unknown ISS values were excluded from the analysis.

See Table 1 for a description of the strengths and limitations of the NEDS database, the proposed analyses, and the codes and variables used.

**Table 1:** NEDS description and analytical methods, including advantages and limitations of the database, proposed analyses, and variables used

Advantages	The NEDS contains a large sample of all ED visits in the US (approximately 20%). It has an Injury Severity Score that is native to the database, and there are multiple methods for assessing injury severity.	
Limitations	Race was not reported as a variable in the database until 2019, which is outside the timeline of the present study. The NEDS relies on the use of external cause ICD-9 codes (E-codes) to indicate law enforcement-related injury.	
Analyses	1) Quantification of the incidence of inpatient admission among ED hospitalizations versus other forms of discharge (*i.e.,* discharge from hospital, discharge against medical advice, died in the ED), over time.*	
	2) Assessment of the change in ISS as a continuous measurement over time, to demonstrate the annual trends in injury severity.	
	3) Evaluation of the change in ISS as a dichotomized measure (based on median ISS) over time, to assess the odds of having an injury severity greater than the median each year.	
Variables	Code	Description
*Firearms or explosives*	E970	Injury due to legal intervention by firearms
	E971	Injury due to legal intervention by explosives
*Gas or poisoning*	E972	Injury due to legal intervention by gas
*Manhandling, cut, or blunt object*	E973	Injury due to legal intervention by blunt object
	E974	Injury due to legal intervention by cutting and piercing instrument
	E975	Injury due to legal intervention by other specified means: blow, manhandling
	E976	Injury due to legal intervention by other unspecified means

*Admittance to inpatient was a proxy for more severe injury, compared to other discharge. The dichotomized outcome was used to compare the NEDS analysis outcomes to the NEISS-AIP outcomes, because the latter does not have an injury score native to the database. This comparison was to confirm internal validity of the study

For all three analyses, crude and adjusted measures were evaluated. Univariate categorical associations were evaluated with odds ratios (ORs) and 95% confidence intervals, while adjusted categorical associations were evaluated with logistic regression and chi square tests. Continuous associations were evaluated with t-tests. Negative binomial and logistic regression models were adjusted for sex and age. P-values < 0.05 were considered significant.

To account for the missing 4^th^ quarter of 2015, all survey weights for 2015 were divided by 0.75, to create an annual estimate for the sample.

### 2006-2015 NEISS-AIP: National Sample of Consumer Product-related Injuries

The National Electronic Injury Surveillance System - All Injury Program (NEISS-AIP) is operated by the US Consumer Product Safety Commission (CPSC) to collect data on product-related injuries, and was extended via interagency agreement to collect data on all injuries for the Centers for Disease Control and Prevention (CDC) [19]. The NEISS-AIP is a nationally representative probability sample of 66 US emergency departments, and contains over 500,000 records, estimating approximately 13 million injury ED visits annually. The NEISS-AIP database contains information on patient demographics (sex, age, race/ethnicity) based on healthcare provider reports, and trained coders review medical record narratives to identify injury, product, diagnosis, disposition, body location of injury, and a narrative description of the injury circumstances.

The 2006 - 2015 NEISS-AIP was queried for all records with intent of injury coded as legal intervention. Legal intervention included injuries sustained during interaction with law enforcement or other legal authorities, but potentially could also include security guards and bar bouncers. Cause of injury was classified as firearms, gas, or manhandling, according to potential lethality. See Table 2 for advantages and limitations of the NEISS-AIP, analyses, and injury definitions.

**Table 2:** NEISS-AIS description and analytical methods, including advantages and limitations of the database, proposed analyses, and variables used

Advantages	The NEISS-AIP relies on trained coders to review patient medical records to build the database. Race is reported as a variable.		
Limitations	The NEISS-AIP is a small sample (66 hospitals) compared with NEDS (953 hospitals). There is no injury severity score native to the database. The NEISS-AIP does not include fatal injuries.		
Analyses	1) Quantification of the incidence of inpatient versus other discharge over time, and by race.*		
	2) Calculation of the risk of inpatient admission given hospitalization, by race by year interaction.		
	3) Assessment of the rate of ED admission and inpatient admission by race over time, per population.		
	4) Evaluation of the rate of ED admission by race over time, per arrests by race, using FBI uniform crime reporting data.		
Variables	Intent	Code	Description
*Firearms or explosives*	INTENT=3 Legal intervention	PCause_C or ICause_C= 18	Precipitating/immediate cause of injury: firearm gunshot
		PCause_C or ICause_C = 19	Precipitating/immediate cause of injury: BB/pellet gunshot
*Gas or poisoning*	INTENT=3 Legal intervention	PCause_C or ICause_C =11	Precipitating/immediate cause of injury: poisoning
		PCause_C or ICause_C =12	Precipitating/immediate cause of injury: inhalation, suffocation
*Manhandling, cut, or blunt object*	INTENT=3 Legal intervention	PCause_C or ICause_C= 7	Precipitating/immediate cause of injury: struck
		PCause_C or ICause_C=8	Precipitating/immediate cause of injury: cut/pierce
		PCause_C or ICause_C=6	Precipitating/immediate cause of injury: fall
		PCause_C or ICause_C=9	Precipitating/immediate cause of injury: overexertion

*Admittance to inpatient was a proxy for more severe injury, compared to other discharge. Because there was no injury score available in the NEISS-AIP database, the dichotomized outcome was used to compare to the NEDS analysis outcomes for internal validity

The NEISS-AIP coded race as White, Black, Asian/Pacific Islander, Native American/Alaskan Native, other, and unknown. Approximately 30% of the race data, 0.07% of the sex data, and 0.01% of the age data was missing, and was thus imputed using fully efficient fractional imputation (FEFI) methods with delete-1 jackknife variance estimation [20]. FEFI methods use multiple known values as a basis for imputing each unknown value. Where comparable age and sex data was available, race was imputed accordingly. Where age and / or sex were missing, they were imputed jointly with race.

Population data were obtained from Statista.com [21], a publicly available research company that specializes in market and consumer data, and arrest data was obtained from the Federal Bureau of Investigation’s (FBI) Uniform Crime Reporting (UCR) Program [22]. Associations between inpatient admission and patient demographics, cause of injury, and year of injury were assessed with ORs and 95% confidence intervals. Chi square tests were used for categorical predictors. P-values of < 0.05 were considered significant.

All original analyses were performed using SAS software, Version 9.4 (SAS Institute Inc., Cary, NC, USA). In order to account for the complex sampling scheme of the NEDS and NEISS-AIP, all analyses except the negative binomial regression were performed using the SURVEY procedures. For the negative binomial regression, which is not included in the SURVEY package, variance was calculated using delete-1 jackknife estimation [23].

## Results

### NEDS Analysis

From 2006 through 2015, there were 529,259 ED admissions for injury related to law enforcement intervention in the NEDS database, averaging approximately 53,000 per year. Men comprised the majority of the admissions (*n*= 454,858; 86%), and the average age was 32.6 years. Most admissions (*n*=438,873; 82.9%) included injuries sustained from being manhandled, cut, or hit with a blunt object. Injuries from being shot by firearm/explosives were less common (*n*=18,726, 3.5%), and gas/poison injuries were the least common mechanism (*n*=5,732, 1.1%). The injury causes were not mutually exclusive, as some admissions included more than one injury mechanism (*i.e.,* some cases may have been both manhandled and shot).

Women admitted to the ED were less likely to be admitted to inpatient than men (Odds Ratio: 0.63, *p*<0.0001). Inpatients were older than other discharge cases (average age 36.5 versus 32.5 years, respectively, *p*<0.0001). The injury severity score (ISS) of inpatient cases was significantly greater than other discharge cases, with an average ISS of 6.4 for inpatients, and 1.7 for other discharge (OR:1.41, *p*<0.0001). Most other discharges were administrative discharges, as opposed to deaths, which accounted for 0.3% of other discharges.

The odds of inpatient admission versus other discharge among ED hospitalizations showed a marginally significant increase over time, of around 2% each year (OR:1.02, *p*=0.057). See Table 3 for details.

**Table 3:** Crude associations for NEDS analysis, odds ratios for inpatient versus other discharge among ED admissions

	Total	Inpatient, *n* (%)	Other Discharge, *n* (%)	*p*-value	Odds Ratio
Total	529,259	22,223 (4.2)	507,036 (95.8)		
Male	454,858 (86.0)	20,118 (90.5)	434,740 (85.8)	< 0.0001	1
Female	74,253 (14.0)	2,105 (9.5)	72,148 (14.2)		0.63 [0.56, 0.70]
Age, mean (standard error)	32.6 (0.09)	36.5 (0.24)	32.5 (0.09)	< 0.0001	
Cause					
Gas/Poison	5,732 (1.1)	91 (0.4)	5,641 (1.1)	< 0.0001	0.37 [0.22, 0.62]
Manhandle/Cut	438,873 (82.9)	14,640 (65.9)	424,233 (83.7)	< 0.0001	0.38 [0.34, 0.42]
Firearm	18,726 (3.5)	5,580 (25.1)	13,147 (2.6)	< 0.0001	12.6 [10.3, 15.3]
Injury Severity (mean, standard error)	1.9 (0.02)	6.4 (0.16)	1.7 (0.01)	< 0.0001	
Year					
2006	48,534	2,149 (9.7)	46,385 (9.1)	0.057	1.02 [1.0, 1.04]
2007	53,558	2,006 (9.0)	51,552 (10.2)		
2008	54,762	2,026 (9.1)	52,736 (10.4)		
2009	55,674	2,176 (9.8)	53,498 (10.6)		
2010	57,311	2,280 (10.3)	55,030 (10.9)		
2011	53,978	2,288 (10.3)	51,691 (10.2)		
2012	54,838	2,384 (10.7)	52,454 (10.3)		
2013	54,219	2,429 (10.9)	51,790 (10.2)		
2014	50,995	2,479 (11.2)	48,516 (9.6)		
2015**	45,390	2,006 (9.0)	43,385 (8.6)		

**2015 data for Q1-Q3 only: sampling weights divided by 0.75 to estimate the entire year

The overall injury severity score (ISS) for the NEDS cohort was 1.9. Seventy percent (*n=*367,668) of all patients injured by law enforcement had an ISS of ≤1. The average ISS for men was higher than for women (1.94 and 1.63, respectively, *p<*0.0001). As expected, cases with gunshot wounds had the highest average ISS (5.3), compared with manhandle/cut (1.8) and gas/poison (0.73). There was a trend toward higher average ISS each year (*p*<0.0001), although average ISS decreased by 4.8% between 2006-7 and by 1.5% between 2011-12. The largest increased percent changes occurred between 2009-10 (5.0%) and 2010-11 (4.7%) The average percent increase in ISS across each year was 0.6%. See Table 4 for details.

**Table 4:** Average continuous Injury Severity Score for risk factors

	Average ISS	*p*-value	
	mean (se)		
Full cohort	1.90 (0.02)		
Sex			
Male	1.94 (0.02)	< 0.0001	
Female	1.63 (0.02)		
Cause			
Gas/Poison	0.73 (0.05)	< 0.0001	
Manhandle/Cut	1.80 (0.02)		
Shot	5.34 (0.27)		
Discharge			
Inpatient	6.40 (0.16)	< 0.0001	
Other	1.70 (0.01)		
Year			Percent change from previous year (%)
2006*	1.86 (0.04)	<0.0001	
2007*	1.77 (0.03)		-4.84
2008*	1.81 (0.03)		2.26
2009	1.81 (0.04)		0.00
2010	1.90 (0.04)		4.97
2011	1.99 (0.04)		4.74
2012	1.96 (0.05)		-1.51
2013	1.97 (0.04)		0.51
2014	1.95 (0.05)		-1.02
2015**	1.95 (0.05)		0.00
Average percent change			0.57

*Injury severity estimated via the ICDPIC program in SAS

**2015 data for Q1-Q3 only: sampling weights divided by 0.75 to estimate the entire year

A survey negative binomial model with delete-1 jackknife variance estimation was used to assess the change in ISS over time, while accounting for sex and age. The result of the model was that the ISS increased by 1.1% each year (*p*<0.0001).

The ISS was dichotomized using the median (1) as a cut point. After adjusting for age and sex, the odds of a patient having an ISS > 1 increased by 1.2% every year.

### NEISS-AIP analysis

From 2006 through 2015, there were 870,779 ED admissions for injury related to law enforcement intervention in the NEISS-AIP database. Men comprised the majority of the admissions (*n*=736,646; 85%), and the average age of all admissions was 32.0 years. Most admissions (*n*=737,345, 85%) were for injuries sustained from being manhandled or cut, compared with shot by firearm (*n*=9,377, 1%), or gassed/poisoned (*n*=3,317, 0.4%). The injury causes were not mutually exclusive. Black patients were the single most frequently admitted race (*n*=358,447, 41.2%), followed closely by White patients (*n*=356,510, 40.9%).

Women were less likely to be admitted as inpatients compared with men (OR: 0.49, *p=*0.02), and inpatient cases were older than other discharge cases (average age 34.8 versus 31.9 years, respectively, *p*<0.0001). Odds of inpatient admission versus other discharge were significantly different across race (*p*=0.03). The odds of inpatient admission versus other discharge among ED hospitalizations showed a significant increase over time, of around 10% each year (OR:1.1, *p*<0.001). See Table 5 for details.

**Table 5:** Crude associations for NEISS-AIP analysis, odds ratios for inpatient versus other discharge

	Total, *n*	Inpatient, *n* (%)	Other Discharge, *n* (%)	*p*-value	Odds Ratio
Total	870,779	30,875 (3.5)	839,904 (96.5)		
Male	736,646	28,285 (91.6)	708,361 (84.3)	< 0.0001	1
Female	134,133	2,590 (8.3)	131,543 (15.6)		0.49 [0.26, 0.94]
Age, mean (se)	32.0	34.8 (0.48)	31.9 (0.36)	< 0.0001	
Race					
Native American / Alaskan Native	9,618	413 (1.3)	9,205 (1.1)	0.04	1.15 [0.48, 2.8]
Asian / Pacific Islander	6,533	141 (0.46)	6,392 (0.76)		0.56 [0.20, 1.6]
Black	358,447	9,904 (32.1)	348,543 (41.5)		0.73 [0.43, 1.23]
Other	139,672	7,027 (22.8)	132,645 (15.8)		1.36 [0.70, 2.62]
White	356,510	13,390 (43.4)	343,120 (40.9)		1
Cause					
Gas/poison	3,317	539 (1.7)	2,778 (0.33)	0.0006	5.36 [1.6, 18.2]
Manhandle/cut	737,345	18,500 (59.9)	718,845 (85.6)	<0.0001	0.25 [0.10, 0.61]
Firearm	9,377	6,527 (21.1)	2,850 (0.34)	<0.0001	78.7 [30.3, 204.3]
Year					
2006	84,383	2,249 (7.3)	82,133 (9.8)	<0.0001	1.10 [1.03, 1.18]
2007	79,730	2,414 (7.8)	77,316 (9.2)		
2008	78,718	1,855 (6.0)	76,863 (9.2)		
2009	83,565	1,824 (5.9)	81,741 (9.7)		
2010	90,914	3,325 (10.8)	87,590 (10.4)		
2011	96,552	2,635 (8.5)	93,917 (11.2)		
2012	98,425	3,948 (12.8)	94,477 (11.2)		
2013	100,645	4,194 (13.6)	96,451 (11.5)		
2014	82,283	4,707 (15.2)	77,576 (9.2)		
2015	75,564	3,724 (12.1)	71,840 (8.6)		

**Fig. 1:**
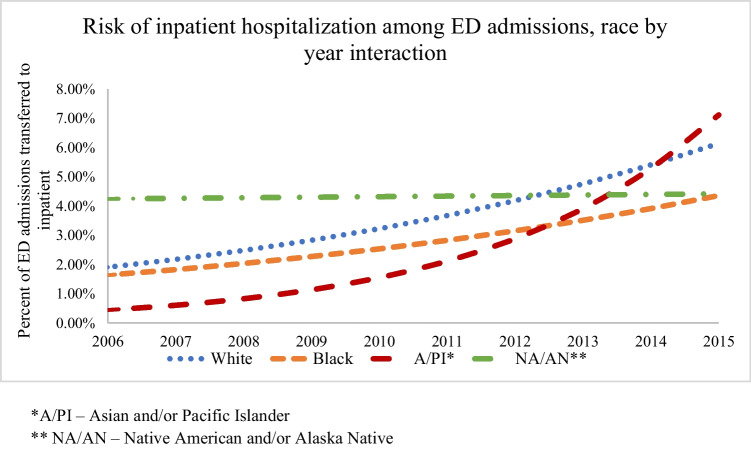
Risk of inpatient admission among ED hospitalizations from law enforcement Use of Force-related injury by race and year interaction, over time. *A/PI – Asian and/or Pacific Islander. ** NA/AN – Native American and/or Alaska Native

In a regression model of the interaction between race and year, the rate of inpatient hospitalization among ED admissions increased for White, Black, and A/PI patients over time, while the rate for NA/AN patients was relatively constant (See Fig. 1). Only specified races were included in the following figures, while “other” race was omitted, which accounted for around 16% of the total study population.

**Fig. 2:**
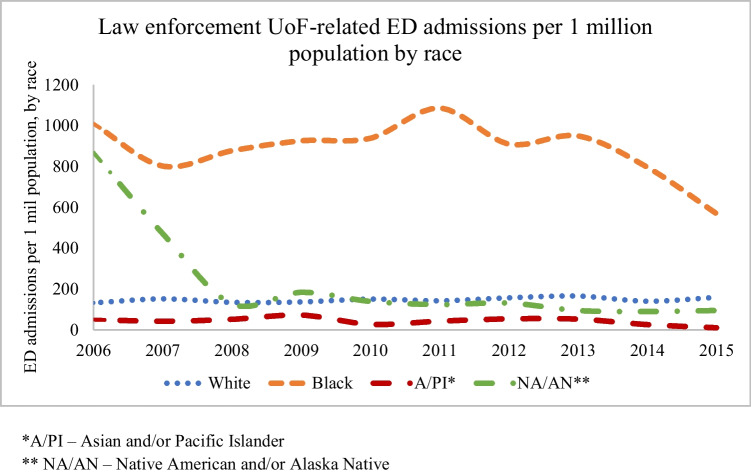
Law enforcement Use of Force-related Emergency Department admissions per 1 million population by race, over time. *A/PI – Asian and/or Pacific Islander. ** NA/AN – Native American and/or Alaska Native

Per population by race, Black people were an average of 6.1 times as likely to be admitted to the emergency department due to UoF as White people, 25.3 times as likely as A/PI people, and 6.2 times as likely as NA/AN people (See Fig. 2).

**Fig. 3:**
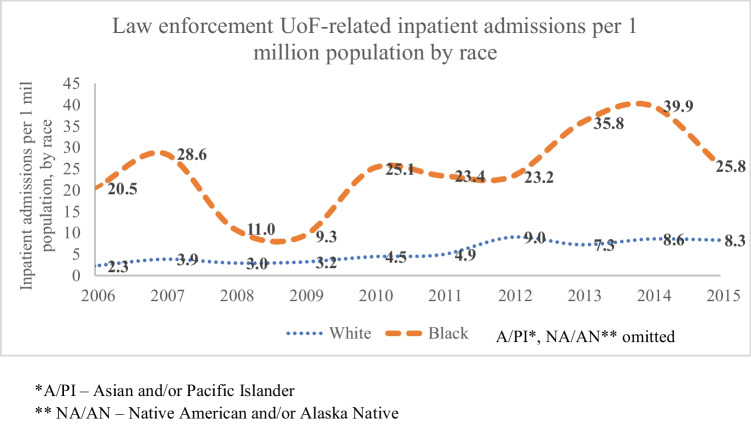
Incidence of inpatient admissions due to law enforcement Use of Force-related injury per 1 million population by race, over time. *A/PI – Asian and/or Pacific Islander. ** NA/AN – Native American and/or Alaska Native

Black ED patients were consistently more likely to be admitted as inpatients versus White patients across all years, per population by race. At the peak rate in 2014, Black inpatient admissions were 4.6 times greater than White inpatient admissions (39.9 patients per population versus 8.6, respectively). Per population, inpatient admissions increased overall during the study period for Black and White people, and the effect was greater for Black people. Due to small cell counts, A/PI and NA/AN samples were omitted from Fig. 3.

**Fig. 4:**
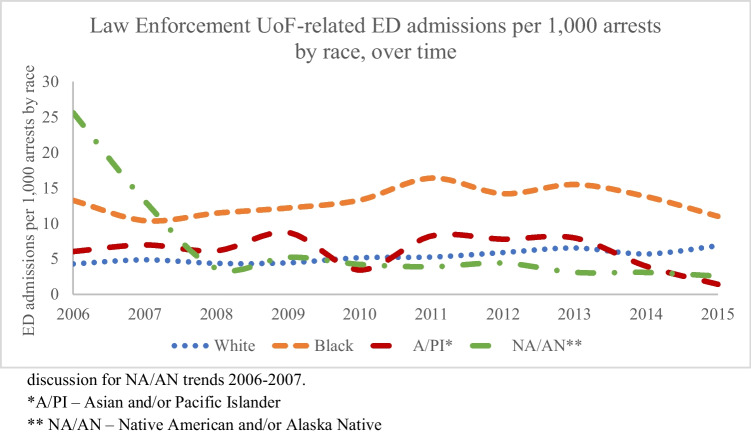
Incidence of law enforcement Use of Force-related emergency department admissions per 1,000 arrests by race, over time, using FBI uniform crime reporting data. *A/PI – Asian and/or Pacific Islander. ** NA/AN – Native American and/or Alaska Native

When adjusted by the annual number of arrests per race using FBI uniform crime reporting data, Black arrestees were on average 2.5 times as likely to be admitted to the emergency department for law-enforcement related injuries as White arrestees, 2.8 times as likely as A/PI arrestees, and 3.1 times as likely as NA/AN arrestees (See Fig. 4). See discussion for NA/AN trends 2006-2007.

## Discussion

The results of the present study indicate that the frequency of law enforcement-related injury has increased significantly between 2006 and 2015 in the United States, a trend that was described previously by Feldman and colleagues [14]. Unique to this study was the finding that the severity of law enforcement-related injury is also increasing over time. Another major study finding was that Black people are injured at higher rates than White people, as previously described by Miller et al. [13], with the added finding in the current study that injury severity differs by race.

Injury severity was measured two ways in this study, firstly as Injury Severity Score (ISS), which is a standardized medical score of traumatic injury, and secondly as the odds of inpatient admission versus other discharge. The significant difference between average inpatient ISS and average other discharge ISS in the NEDS confirmed that inpatient versus other discharge was an appropriate proxy for injury severity and was the measure of injury severity for the NEISS-AIP analysis, because the database did not contain a built-in injury severity measure. Injury severity due to law enforcement Use of Force (UoF), when accounting for age and sex, increased by 1.1% each year. The odds of inpatient admission versus other discharge increased annually by 2% in the NEDS, and 10% in the NEISS-AIP. The risk of UoF injury per population increased during the study period for White, Black, and A/PI patients, and stayed relatively constant for NA/AN patients.

Every metric of injury frequency and severity examined in the current analysis indicated a disproportionate effect of UoF among Black people. More than 40% of the ED admissions from law enforcement-related injury in this study were among Black people, which is a 43% overrepresentation of Black arrests in the US (28% of the total), and a 230% overrepresentation of the Black population (12% of the US total population) [24, 25]. Black people were 6 times more likely (per population) to be admitted to the ED for UoF injury than White and NA/AN people, and 25 times more likely than A/PI people. Using inpatient admission as a proxy for injury severity, there was also evidence that Black people are more likely to sustain more severe UoF-related injuries, as they were 4.6 times more likely to be admitted to inpatient than White people, per population. Per arrest using FBI arrest data, Black arrestees were 2.5 times more likely to be admitted to the ED than White arrestees, 2.8 times as likely as A/PI arrestees, and 3.1 times as likely as NA/AN arrestees. The results of this study indicate that Black people are more frequently and more severely injured by law enforcement than White, A/PI, or NA/AN people, and that both the rate and severity of injury have increased over time.

The 2014 Ferguson protests following the law enforcement killing of Michael Brown, as well as the 2020 nationwide protests following the law enforcement killings of George Floyd and Breonna Taylor, shone a spotlight on the lack of accountability of law enforcement violence, and especially the racially disparate outcomes of law enforcement violence. The FBI released its UoF database participation data after these protests, but the prevalence and incidence of law enforcement UoF remains unknown; less than 50% of agencies are voluntarily participating in the database, and law enforcement continues to control the narrative around UoF. The results of the current study provide support for the base claims of the protests, which is that law enforcement violence systematically targets Black people.

A law enforcement officer’s decision to use force against a suspect depends on the officer’s perceived risk to themselves, the suspect, and others. An officer is authorized to use the level of force that they determine to be necessary to apprehend a subject. The use of force continuum ranges from officer presence, verbal commands, empty-hand techniques (the use of bare hands, with no weapons), less-lethal techniques (TASER shocks, pepper spray, baton strikes), and finally lethal force [26]. The ideal use of force is only what is necessary to subdue and detain a possible suspect.

*Excessive UoF* is a term used to describe a systematically high rate of law enforcement UoF across a population, relative to other populations, indicating that law enforcement is using force against civilians liberally. Defining and measuring excessive UoF is challenging, as some percentage of injuries result from UoF is proportionate, and not excessive. While there is no known base rate of expected injury from appropriate UoF, the finding in the present study that injury rate and severity has increased over time provides a strong indication of increasing violence of law enforcement tactics, and evidence of systematic increase in UoF by law enforcement on the US population. The explanation for this phenomenon is uncertain, however, several authors have described an association between an increase in civilian fatalities and increased “militarization” of law enforcement tactics, secondary to an influx of military equipment and training of law enforcement [27, 28]. The result of militarization is that law enforcement personnel are more likely to view suspects as “the enemy,” rather than as members of their own community. Following the 9/11 terrorist attacks, counterterrorism laws and funding allowed for unprecedented militarization of domestic law enforcement. It is reasonable that this shift in tactics would explain the increase in the rate and severity of injuries during the study period, and support the assertion that there has been an increase in the rate of excessive UoF.

There are alternative explanations for the observations in the present study that must be considered. One explanation is that UoF is proportional to increased rates of violent crimes. No correlation has been found between crime rates and law enforcement related deaths in the 50 largest cities in the US, however [29]. It is reasonable that this lack of association extends to UoF-related injuries as well.

Another explanation is increased surveillance for law enforcement-related hospital admissions during the timeframe of the study, such that hospital staff were coding for law enforcement external causes (*i.e.,* ICD-9 E-codes) more often over time. Although plausible, this explanation doesn’t account for the consistent increase in frequency, and more critically, *severity* of law enforcement-related injuries, nor does it account for the disparity in trend between the races.

Given the lack of a more likely alternative explanation, it is thus most likely that an increase in excessive UoF best explains our findings. While the trend of increased injury frequency and severity was observed across all US ED admissions over the study period, the rate and severity were highest for Black people. When adjusted by the rate of arrests for all offenses, injuries to Black arrestees were 2.5 times more likely than White arrestees, when using FBI arrest data to determine the denominator. Thus, increased rates of injury to Black people are not due to increased interaction between law enforcement and Black people compared to other races, but rather that law enforcement are more likely to use force against Black people per interaction, and for that force to be more violent, and more likely to result in injury and ED and inpatient admission.

There are several limitations to consider in the current study, primarily due to the nature of the databases used. The NEDS is a more robust database than the NEISS-AIP, as it uses nearly 953 hospitals to provide national weighted ED estimates, while the NEISS-AIP uses only 66 hospitals for the same estimates, which may be too small of a sample to represent nationwide racial trends accurately or precisely. It is likely that the disproportionate sampling accounts for the differences in counts across similar variables (*i.e.,* the total number of subjects in the NEDS was 529,259 versus 870,779 in the NEISS-AIP). The imputed data for the fourth quarter of 2015 likewise may not accurately represent seasonal trends in the year. Additionally, security guards and bar bouncers were included as law enforcement in the NEISS-AIP, which comprise an unknown (but likely small) percentage of the total number of hospitalizations due to UoF. This population should not be excluded from the study, however, as security guards and bouncers use similar tactics as police to restrain subjects. Thus, the hospitalizations of those injured by security guards or bouncers likely comprise a trivial proportion of the populations described in the present study.

The race variable within the NEISS-AIP was missing for 30% of the sample, which was accounted for in the imputation analysis using fully efficient fractional imputation (FEFI). FEFI is a well-described and validated statistical method for estimating missing data [30]. Compared to the more traditional multiple imputation methodology, FEFI provides a less-biased estimate of the sampling variation, and thus more accurate confidence intervals. Race was imputed individually according to observed age and sex, or jointly with age and sex where those data were also missing (0.07% and 0.01% of the sample respectively). A comparison between imputed and unimputed data showed similar population estimates, and as expected, a decrease in the standard error associated with the larger sample size available in the imputed data. Small cell counts resulted in poor approximation for Asian and Pacific Islander, as well as Native American and Alaska Native samples. “Other” race was the third most common race (16%) and may have been a catchall for people of mixed race. In the first two years of the study (2006-2007), the category of NA/AN had the second highest proportion of UoF injuries relative to the population and arrest rates (see Figs 2 and 4). An increase in “other” race patients starting in 2008 correlated proportionately with a decrease in NA/AN patients the same year, supporting the theory that patients previously categorized as NA/AN were more likely to be categorized as “other” race beginning in 2008.

Another potential limitation, resulting in underestimation of the study outcomes, is that cases resulting in death that occurred while outside of the hospital were not included. This population would have included more serious injuries than are represented by the patients who survived to be brought to the ED. Another potential source of undercounting is individuals who were injured by law enforcement but not brought to the ED and were either not treated for their injuries or were treated for injuries at another facility such as jail or at an outpatient facility. This population would likely comprise less severe injuries than the sample that is brought to the ED.

## Conclusions

The findings of the present study demonstrate that US law enforcement are injuring civilians both more frequently and more severely over time. Black people are disproportionately more likely to be injured by law enforcement, at a rate of at least 6 times that of other races. When Black people are injured by law enforcement, they are more than 4 times more likely than White people to be admitted to inpatient care. When adjusted by arrest rate by race using FBI arrest data, Black arrestees are 2.5-3.1 times more likely to be injured by law enforcement than White, NA/AN, or A/PI arrestees. In the absence of plausible alternative explanations for these trends, the remaining explanation is that US law enforcement personnel are becoming more violent, which is systematically injuring Black people more frequently and more severely than any other race.

The development of a nationwide database of law enforcement-related injuries and fatalities with required participation from law enforcement agencies will allow for more accurate accounting of the effects of UoF on the US population, and help identify racial, geographic, and other parameters associated with excessive UoF. Re-direction of law enforcement training away from mandatory confrontation in all circumstances, and toward de-escalation when feasible will decrease both risk and severity of preventable injuries.

## Data Availability

Raw data were generated using the Nationwide Emergency Department Sample (NEDS), as a part of a family of databases developed by the Healthcare Cost and Utilization Project by the Department of Health and Human Services, and sponsored by the Agency for Healthcare Research and Quality, as well as the National Electronic Injury Surveillance System - All Injuries Program (NEISS-AIP), from the Consumer Product Safety Commission, and expanded by the Centers for Disease Control and Prevention. Derived data supporting the findings of this study are available from the corresponding author E.M.F.S. on request.
